# Impact of HVAC-Systems on the Dispersion of Infectious Aerosols in a Cardiac Intensive Care Unit

**DOI:** 10.3390/ijerph17186582

**Published:** 2020-09-10

**Authors:** Larisa Anghel, Cătălin-George Popovici, Cristian Stătescu, Radu Sascău, Marina Verdeș, Vasilică Ciocan, Ionela-Lăcrămioara Șerban, Minela Aida Mărănducă, Sebastian-Valeriu Hudișteanu, Florin-Emilian Țurcanu

**Affiliations:** 1 Internal Medicine Department, Grigore T. Popa University of Medicine and Pharmacy, 700503 Iași, Romania; larisa.anghel@umfiasi.ro; 2 Cardiology Department, Cardiovascular Diseases Institute, Prof. Dr. George I.M. Georgescu, 700503 Iași, Romania; 3 Building Services Department, Faculty of Civil Engineering and Building Services, Gheorghe Asachi Technical University, 700050 Iaşi, Romania; catalin.popovici@tuiasi.ro (C.-G.P.); marina.verdes@tuiasi.ro (M.V.); vasilica.ciocan@tuiasi.ro (V.C.); sebastian.hudisteanu@tuiasi.ro (S.-V.H.); emilian-florin.turcanu@tuiasi.ro (F.-E.Ț.); 4 Physiology Department, Grigore T. Popa University of Medicine and Pharmacy, 700503 Iași, Romania; ionela.serban@umfiasi.ro (I.-L.Ș.); minela.maranduca@umfiasi.ro (M.A.M.)

**Keywords:** COVID-19, airborne transmission, intensive care unit, air conditioning systems, hospital-acquired infections, risk factors

## Abstract

At the end of 2019, a variation of a coronavirus, named SARS-CoV-2, has been identified as being responsible for a respiratory illness disease (COVID-19). Since ventilation is an important factor that influences airborne transmission, we proposed to study the impact of heating, ventilation and air-conditioning (HVAC) with a variable air volume (VAV) primary air system, on the dispersion of infectious aerosols, in a cardiac intensive care unit, using a transient simulation with computational fluid dynamics (CFD), based on the finite element method (FEM). We analyzed three scenarios that followed the dispersion of pathogen carrying expiratory droplets particles from coughing, from patients possibly infected with COVID-19, depending on the location of the patients in the intensive care unit. Our study provides the mechanism for spread of infectious aerosols, and possibly of COVID-19 infection, by air conditioning systems and also highlights important recommendations for disease control and optimization of ventilation in intensive care units, by increasing the use of outdoor air and the rate of air change, decreasing the recirculation of air and using high-efficiency particulate air (HEPA) filters. The CFD-FEM simulation approach that was applied in our study could also be extended to other targets, such as public transport, theaters, philharmonics and amphitheaters from educational units.

## 1. Introduction

Hospital-acquired respiratory tract infections, also known as nosocomial respiratory tract infections, have proven to be a challenge and sometimes a tragic problem for the practitioner. They can be caused by a variety of viruses that spread through three different routes: contact (direct and indirect), droplet and aerosol transmission [1,2]. Transmission by direct contact means that, from an infected patient to a susceptible person, the virus spreads through contaminated hands, for example. When the virus is transmitted through intermediate objects, such as fomites, we are talking about indirect virus transfer. The virus can also be transmitted via the air, droplets or aerosols [3,4,5]. Pulmonary activities such as coughing [6,7], breathing [8], sneezing [8] or talking [9], are sources of bio-aerosols that can have respiratory tract infections pathogens [7,8,9]. Large droplets (>10 microns), formed especially from coughing and sneezing, fall on surfaces and objects not further than 1–2 m from the infected patient [3,10]. People can catch the infection directly by standing within 1–2 meters of an infected person and breathing in these droplets. When people are standing within 1–2 meters of an infected patient or when they touch their mouth, nose or eyes after they touched the contaminated surfaces or objects, they can catch the infection [10]. Droplets that evaporate (10 microns droplets evaporate in 0.2 s) and desiccate, form small particles (droplet nuclei or residue). Aerosols are small particles (<5 microns), with a slow velocity and may remain suspended in the air for hours and can be transported long distances [1,4,11]. 

Respiratory viruses spread via these three routes depends on environmental factors, such as humidity or temperature and crowding of people, and also on host factors such as receptor distribution all over the respiratory tract [5,11]. 

Airborne transmission is responsible for more than one third of hospital-acquired infections [12,13]. This dangerous threat of airborne infection to human health has been reiterated by the severe acute respiratory syndrome (SARS) in 2002–2003 [14,15], pandemic influenza A virus subtype H1N1 (A/H1N1) in 2009 [16], Middle East Respiratory Syndrome (MERS) in 2012 [17] and the ongoing pandemic of coronavirus disease 2019 (COVID-19), caused by severe acute respiratory syndrome coronavirus 2 (SARS-CoV-2) [18]. 

Since 31 December 2019, when the Wuhan Municipal Health Commission reported 27 cases of viral pneumonia, there was a rapid spread around the country and the entire world. This disease was named Coronavirus Disease 2019 (COVID-19) by the World Health Organization. On 19 July 2020, the number of confirmed cases reached 14,007,791, with 597,105 deaths worldwide, according to the World Health Organization [19,20,21]. Coronaviruses have a small size of 70–120 nanometers [20,21,22] and SARS-CoV-2 remains active up to three hours in indoor air and up to three days on room surfaces, unless there is specific cleaning [20,21,22,23,24,25,26]. Because SARS-CoV-2 has such a small size, it can be carried by airflows, either in the room or in the extract air ducts of ventilation systems, and it can travel long distances [25]. It is known that SARS-CoV-2 is mainly transmitted via respiratory droplets released from infected persons [19]. Current studies suggest that COVID-19 may be transmitted through droplets from patients with mild symptoms and even from asymptomatic ones [22], but not through long-range inhalation of aerosols, as in the case of tuberculosis or measles. However, considering that many respiratory infectious agents have a short-range aerosol transmission, this cannot be excluded in the case of SARS-CoV-2 infection, especially in poorly ventilated spaces or in crowded medical departments [19]. According to the World Health Organization, during “medical aerosol generating procedures,” an airborne transmission of SARS-CoV-2 infection, through droplet nuclei (aerosols), can be encountered [26]. Until now, airborne transmission of SARS-CoV-2 has been demonstrated only in a few experimental studies, and not for humans. In the absence of aerosol-generating procedures, further studies are needed in order to determine the role of aerosols in SARS-CoV-2 transmission. Considering the small number of studies that evaluated the SARS-CoV-2 transmission in medical departments, we aimed to evaluate the impact of heating, ventilation and air-conditioning (HVAC) systems on the dispersion of infectious aerosols, and possibly of COVID-19 transmission, in a cardiac intensive care unit. We chose this coronary intensive care unit due to the increased addressability of patients with serious health problems, who may require orotracheal intubation and mechanical ventilation. 

Airborne transmission occurs more frequently in indoor environments where people spend over 90% of their time [27], and it involves generation, transformation, transport and finally inhalation of aerosols. Ventilation is one of the most important factors that influence airborne transmission [10]. Thus far, few studies have demonstrated an association between ventilation of buildings and the transmission of airborne infections [28,29,30]. Furthermore, there are no sufficient studies regarding the minimum ventilation requirements in hospitals, and especially in intensive care units, regarding the airborne transmission infections. Air purifiers with high-efficiency particulate air (HEPA) filters may be a complementary way for removal of SARS-CoV-2 aerosols in healthcare settings. It is known that the disease spreads through droplet transmission, but studies are ongoing on what can stop or aid the transmission, despite social distancing and a proper disinfection [31]. Maintaining distance from an infected person might be insufficient and increasing the ventilation might be useful, considering that this will remove more particles [20]. 

Because ventilation is one of the most important factors that influence airborne transmission, it is necessary to study the dispersion, heat and mass transfer of pathogen carrying expiratory droplets for better infection control strategies. Patients hospitalized in the cardiac intensive care units are at high risk of developing major infections [32], which sometimes are associated with lethal clinical consequences and higher costs, due to prolonged hospitalizations and more frequent readmissions [33]. Reducing the risk of hospital-acquired respiratory tract infections associated with air conditioning systems is a key priority for improving intensive cardiac therapy. Considering that patients from cardiac intensive care units are older and have other comorbidities that further increases their infection risk, COVID-19 infection represents a management challenge in these cases [34]. 

Considering the worldwide spread of the SARS-CoV-2 infection, the large number of infected people and also deaths, we consider that it is critical to understand the modes of transmission of SARS-CoV-2, in order to develop effective prevention and control strategies. In this clinical and epidemiological context, we proposed to study the impact of heating, ventilation and air-conditioning with a variable air volume (VAV) primary air system on the dispersion of infectious aerosols, and possibly on COVID-19 transmission, in a cardiac intensive care unit.

## 2. Materials and Methods 

### 2.1. Room Model

The study was performed in a Cardiac Intensive Care Unit with an increased addressability of patients with serious health problems. The cardiac intensive care unit room contains seven beds with the following geometry: Length × Width × Height = 13.00 m × 6.80 m × 2.75 m. The area of the room is around 83 m^2^ and the volume is 229 m^3^. The intensive care unit is placed in the third wind zone and the third climatic zone of Romania, with an outdoor temperature during winter of −18 ℃ (the design temperature). In order to study the dispersion of particles from coughing, influenced by air conditioning systems, we used the Computational Fluid Dynamics (CFD) approach, based on the finite element method (FEM). The CFD models are the most used in order to improve the indoor air quality, but there still are two major challenges: turbulence modeling and experimental validation. Data acquisition was performed using a TESTO 480 equipment with an indoor air quality (IAQ) sensor for airflow and particle concentration. Additionally, we used a TESTO DiSCmini particle counter to study the particle dispersion characteristics for PM_10_ and PM_2.5_. We evaluated these two parameters to see the indoor air quality from the intensive care unit, with the existing heating system. Following the measurements, we observed values above the upper limit in case of PM_2.5_ (the mean value was 29 µg/m^3^) and at the upper limit of normal for PM_10_ (the mean value was 48 µg/m^3^). We used these results to define the particle concentration when we made the CFD simulation for the existing heating system. The software used for CFD analysis was ANSYS FLUENT 2020 R1, with Elastic Licensing Number 1078891, and we performed a three-dimensional (3D) numerical study of the indoor air. 

**Figure 1:**
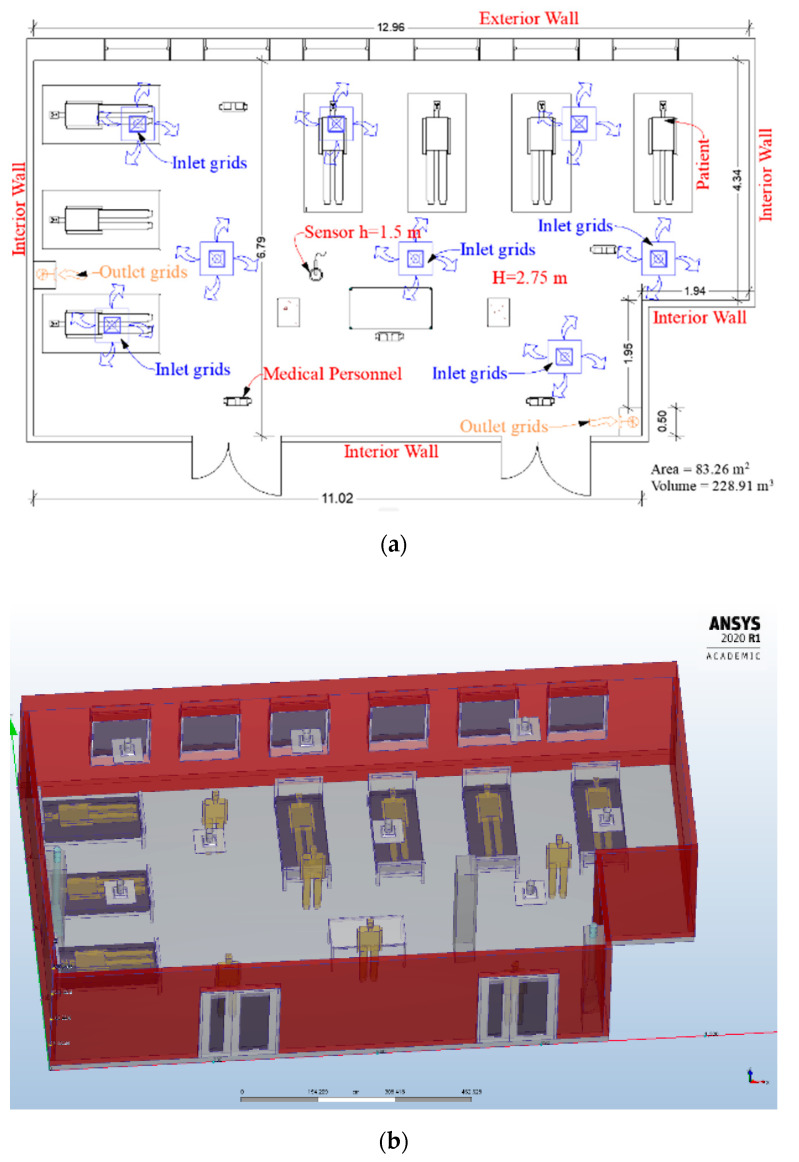
(**a**) Floor plan of the cardiac intensive care unit with the proposed heating, ventilation and air conditioning system (HVAC); (**b**) the proposed three-dimensional (3D) model of the cardiac intensive care unit from Ansys 2020 R1 software.

The heating system from the cardiac intensive care unit used static heaters, but, unfortunately, two years ago it was severely damaged by a fire. Considering the recommendation of the Federation of European Heating, Ventilation and Air Conditioning Associations (REHVA) [10] and the needs for thermal comfort and indoor air quality, it was changed with a heating, ventilation and air conditioning system (HVAC) (Figure 1). The HVAC system has seven inlet grids with the dimension of 600 × 600 mm and two outlet grids of 700 × 300 mm, placed in the lower third of the room height. The total airflow rate is 8400 m^3^/h and the system work with 100% fresh air supply. The airflow rate for each inlet grid is 1200 m^3^/h and for the outlet grids is 85% of the total flow, in order to maintain an overpressure of 12.5 Pa, compared to the connecting rooms. The HVAC system has the following operating parameters: temperatures 24–26 ℃ and humidity between 30 and 60%.

We measured the indoor and outdoor temperatures during winter and also taking into account the envelope parameters and the thermal resistance of the walls and windows, we had the boundary conditions to develop the model for computational fluid dynamics. 

### 2.2. Modeling Turbulence

Based on literature studies, we selected three different turbulence models for CFD analysis and for the validation of the model: the standard k-ε model, SST-k-ω and RNG-k-ε.

The most used models for indoor climate modeling are k-ε and k-ω. These two models fall into the Reynolds-Averaged Navier-Stokes (RANS) model category. The standard k-ε model is the most used today. The basic idea was to develop an equation for ε, including reasonable approximations for coefficients and to solve the equation of k with a similar equation.

The equations are expressed as follows:

(1) $$\frac{\partial (\rho u_{i})}{\partial t}+\frac{\partial (\rho u_{i}u_{j})}{\partial x_{j}}=\frac{\partial }{\partial x_{j}}\left[(\mu +\mu_{t})\left(\frac{\partial {\bar{u}}_{i}}{\partial x_{j}}+\frac{\partial {\bar{u}}_{j}}{\partial x_{i}}\right)\right]-\frac{\partial p}{\partial x_{j}}$$

where:

(2) $$\mu_{t}=C_{\mu }k^{2}/\varepsilon$$

The kinetic energy equation is of the form:

(3) $$\rho \frac{\partial k}{\partial t}+\rho u_{j}\frac{\partial k}{\partial x_{j}}=\frac{\partial }{\partial x_{j}}\left[(\mu +\mu_{t}/\sigma_{k})\frac{\partial k}{\partial x_{j}}\right]+P_{k}-\varepsilon$$

Dissipation rate:

(4) $$\rho \frac{\partial \varepsilon }{\partial t}+\rho u_{j}\frac{\partial \varepsilon }{\partial x_{j}}=\frac{\partial }{\partial x_{j}}\left[(\mu +\mu_{t}/\sigma_{\varepsilon })\frac{\partial \varepsilon }{\partial x_{j}}\right]+C_{\varepsilon 1}\frac{\varepsilon }{k}P_{k}-C_{\varepsilon 2}\frac{\varepsilon^{2}}{k}$$

where the closing coefficients are defined as follows: C_ε1_ = 1.44; C_ε2_ = 1.92; C_μ_ = 0.09; σ_k_ = 1.0; σ_ε_ = 1.3.

In a more recent version of the k-ε model a group renormalization theory (RNG) is used [35]. The RNG model proposes a modification of the transport equation where the source term is solved as follows:

(5) $$S_{\varepsilon }=\frac{\varepsilon }{k}(C_{\varepsilon 1}P_{}-C_{\varepsilon 2}D)-R$$

In the standard model k-ε, the term R is missing; the term R is defined as follows:

(6) $$R=\frac{C_{\mu }\eta^{3}(1-\eta /\eta_{0})}{1+\beta \eta^{3}}\frac{\varepsilon^{2}}{k}$$

where β and η_0_ are constant and have the values 0.015 and 4.38, respectively.

The effect that the correction has on the standard model k-ε, the introduction of this term R is the ability to react to the rapid change of voltage and the simplification of the curvature of current lines.

The constants in the turbulent transport equation have the following values: C_μ_ = 0.0845, σ_k_ = 0.718; σ_ε_ = 0.718, C_ε1_ = 1.42; C_ε2_ = 1.68.

The RNG k-ε model is better to model high and low Reynolds numbers in the same flow. This is the reason why this turbulence model is preferred for ventilation, air quality problems and airborne infection in healthcare units. Additionally, in our study, we observed the validation of the model for the RNG k-ε turbulence model.

The k-ω model was developed by Kolmogorov [36]. It has several advantages over the k-ε model, being able to calculate the unfavorable effects of pressure gradients. The Shear Stress Transport model (SST-k-ω) was first stated by Menter [37]. The equations describing the SST-k-ω model is based on both components of the k-ε and k-ω turbulence model.

Kinetic energy equation:

(7) $$\frac{\partial \rho k}{\partial t}=\tau_{ij}^{}\frac{\partial u_{i}}{\partial x_{j}}-\beta *\rho k\omega +\frac{\partial }{\partial x_{j}}\left[(\mu +\sigma_{k}\mu_{t})\frac{\partial k}{\partial x_{j}}\right]$$

Dissipation rate:

(8) $$\frac{\partial \rho \omega }{\partial t}=\frac{\gamma }{\nu_{t}}\tau_{ij}^{}\frac{\partial u_{i}}{\partial x_{j}}-\beta *\rho \omega^{2}+\frac{\partial }{\partial x_{j}}\left[(\mu +\sigma_{k}\mu_{t})\frac{\partial \omega }{\partial x_{j}}\right]-2\rho (1-F_{1})\sigma_{\omega 2}\frac{1}{\omega }\frac{\partial k}{\partial x_{j}}\frac{\partial \omega }{\partial x_{j}}$$

where:

(9) $$2\rho (1-F_{1})\sigma_{\omega 2}\frac{1}{\omega }\frac{\partial k}{\partial x_{j}}\frac{\partial \omega }{\partial x_{j}}$$

represents the cross-diffusion rate

(10) $$P_{\omega }=\gamma \frac{\omega }{k}\tau_{ij}^{}\frac{\partial u_{i}}{\partial x_{j}}$$

represents the production term

(11) $$\nu_{t}=\frac{k}{\omega }.$$

The model constants have the following values: $\sigma_{k,1}=1.176$, $\sigma_{k,2}=1$, $\sigma_{\omega ,1}=2$, $\sigma_{\omega ,2}=1.168$, α_1_ = 0.31, β_i,1_ = 0.075, β_i,2_ = 0.0828.

**Figure 2:**
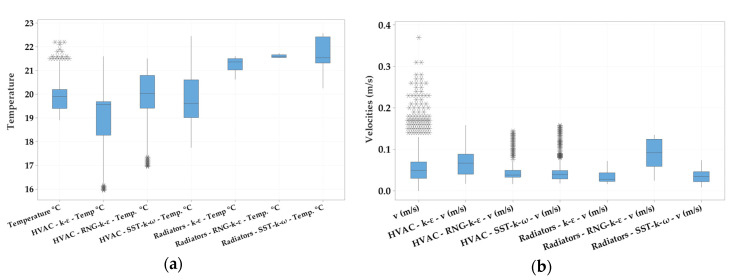
Boxplot of temperature (**a**) and air velocities (**b**) variation from indoor air quality sensors, static heaters and heating, ventilation and air conditioning (HVAC) system, for the three turbulence models (standard k-ε, SST-k-ω and RNG-k-ε).

Subsequently, we compared the values for temperature and air velocities measured with the indoor air quality sensor with those from numerical simulations, and we observed the validation of our model only for the RNG-k-ε turbulence model, the differences between these values being below 10% (Figure 2). Considering the validation of our model for the RNG-k-ε turbulence model, the results presented in our study are only for this turbulence model. 

### 2.3. Time Discretization

In addition to the steady-state analysis, we used a transient analysis to see the time dispersion of particles from coughing, influenced by air conditioning systems. The proper selection of cough simulation and particle advancement time steps are very important and also challenging. It depends on the nature and turbulent characteristics of the flow and also on the space grid refinement [30]. For an accurate dispersion of the droplets in time, we considered three criteria: the droplets did not cross more than one grid cell for each time step; the time step was smaller than that of the eddy lifetime and crossing time. To investigate the behavior of the cough droplets induced from the patients and influenced by HVAC, the discrete phase model was used in ANSYS FLUENT 2020 R1, in order to predict the trajectory of the droplets. Lagrangian simulations can predict the trajectory of a particle through the airflow and also permits the simulation of airflow with particles, being appropriate for solid and liquid particles from airflow [38]. In order to analyze the particle motion within the room, we used the discrete phase model, based on the Lagrangian discrete random walk model. This can be made by integrating and equating the particle inertia with the force that acts on the droplet in the Lagrangian reference [39]. In our case, the equation for the z-direction is:

(12) $$\frac{du_{d}}{dt}=F_{D}\left(U-U_{d}\right)+g_{z}\frac{\left(\rho_{d}-\rho \right)}{µ}+F_{z}$$

where:

$F_{D}\left(U-U_{d}\right)$ is the drag force per unit droplet mass;

$U_{d}$ is the velocity of the droplet;

$\rho_{d}$ is the density of the droplet;

$g_{z}$ is the force of gravity of the droplet in z direction;

µ is the molecular viscosity

$F_{z}$ is an additional force. 

**Figure 3:**
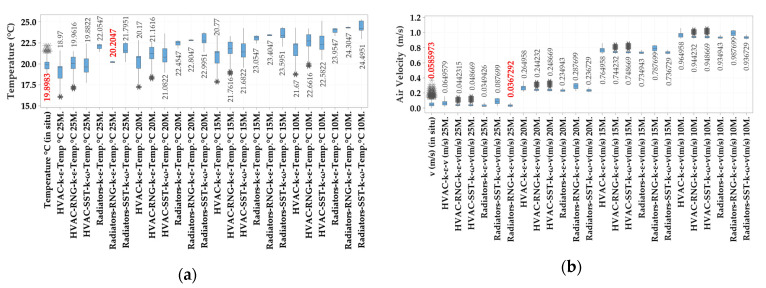
Results for mesh dependents (**a**) temperature and (**b**) air velocities from indoor air quality sensors, static heaters and heating, ventilation and air conditioning (HVAC) system, for the three turbulence models (standard k-ε, SST-k-ω and RNG-k-ε) and four cases of discretization (10, 15, 20, 25 M).

Using an HP Platform Z840, with two Xeon Processor E5-2690 v4, we performed the simulation on this model, which lasted over 672 h (4 weeks). We made an initial discretization of 10 million elements. Afterwards, we increased the number of meshing elements to 15, 20 and 25 million, until the difference between the models was under 1% variation (Figure 3).

### 2.4. Boundary Conditions

In order to validate our study, ANSYS FLUENT 2020 R1 was used. The validation process consists in observing the reliability and accuracy of the ANSYS Fluent CFD program used to simulate a numerical case by comparing it with experimental data. Additionally, we used the same boundary conditions that were used during the experiment.

**Figure 4:**
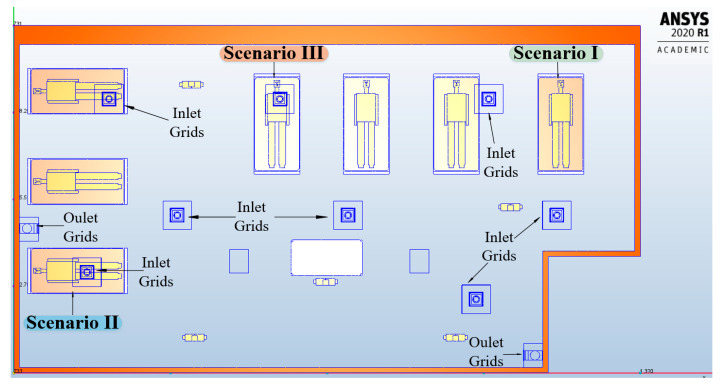
The three scenarios for the HVAC system, according to the location of the patients in the intensive care unit.

For the HVAC system, we analyzed three scenarios that followed the dispersion of pathogen carrying expiratory droplets particles from patients admitted to the intensive care unit and possibly infected with COVID-19. Coughing and sneezing are the most important processes of virus transmission because of the highest droplet concentration [40]. Considering that coughing is the common symptom for most respiratory tract infections, in our study we used boundary conditions for the coughing process. The characteristics of coughed droplets for the CFD simulation were selected by taking into account the existing data from the literature [40,41,42,43,44]. We used seven manikins as patients and four manikins as medical personnel. The dimensions of the manikins were similar to those of a real human body, and the mouth opening area of the manikin was 3.5 cm^2^ [41]. We observed the dispersion in time and space of the particles from coughing, influenced by the HVAC system, depending on the location of the patients in the intensive care unit: in the right side, left side and in the middle part of the intensive care unit (Figure 4).

The advantage of CFD simulations is that they are inexpensive compared to experiments, but they need accurate flow-thermal boundary conditions, which are very important for the prediction of disease transmission. The most important boundary conditions for expiration are coughing flow rate and jet direction, mouth opening areas, temperature and size distribution of the virus droplets [41,42,43,44]. In all three proposed scenarios, the manikin coughed three times, and the duration of one act of coughing was 0.3 s. The CFD simulations for prediction of disease transmission used the same velocity, cough flow rate and mouth opening area. The particle size was between 2.5 µm and 200 µm. In Table 1 we presented the boundary conditions used in our CFD simulations.

**Figure 5:**
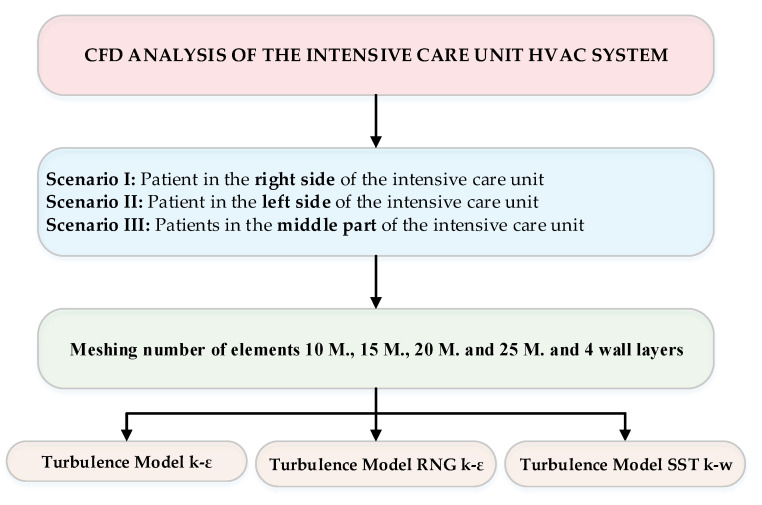
CFD analysis scheme for the cardiac intensive care unit simulations.

After establishing the geometric model and the competition of the boundary conditions, we made the following simulations (Figure 5). 

## 3. Results

### 3.1. Particle Dispersion for Static Heaters and HVAC

**Figure 6:**
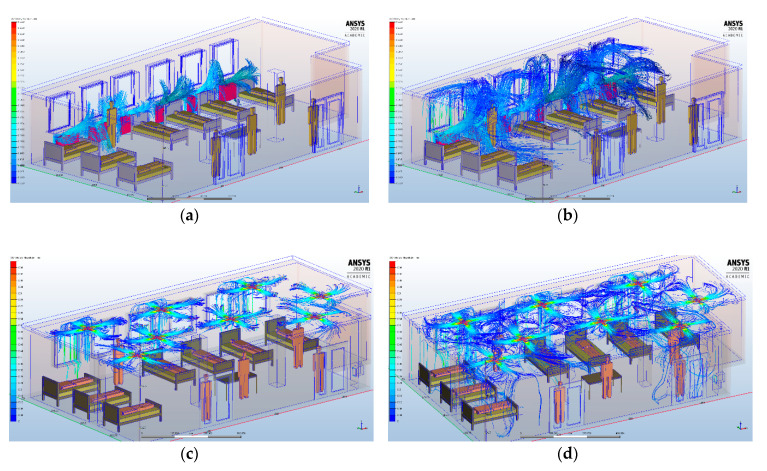
Transient analysis of particles dispersion with no coughing and breathing events of the patients for static heaters at (**a**) 2 s and (**b**) 5 s, and the air inflow dispersion for heating, ventilation and air conditioning systems at (**c**) 2 s and (**d**) 5 s.

Initially, we analyzed the particle dispersion generated by the heating system that was present before the fire: static heaters. Subsequently, we performed the same analysis for the heating system that will be put into use after rehabilitation, namely HVAC. We wanted to highlight the difference between these two heating systems, in terms of dispersion of infectious aerosols (Figure 6).

For the simulation performed with static heaters, we defined a number of 6000 particles with dimensions between 2.5–200 micrometers, located in the maximum area of influence of the air currents generated by the static heaters. In the case of heating, ventilation and air conditioning systems, we also used a number of 6000 particles with the same dimensions, which were split equally to the seven inlet grids. In a transient analysis of particle dispersion at 3 s after the air currents are generated by each heating system, we observed the distribution of particles near the static heaters, without them spreading throughout the intensive care unit. With the HVAC system, the particles are distributed on all four directions at the ceiling, foreshadowing a uniform distribution throughout the room. Although the HVAC system may initially appear to achieve a faster and more uniform distribution of infecting particles, this is counterbalanced by the possibility of using HEPA filters, which are an adjunctive means for the decontamination of SARS-CoV-2 aerosols in healthcare settings [45,46,47]. 

### 3.2. Scenario 1

**Figure 7:**
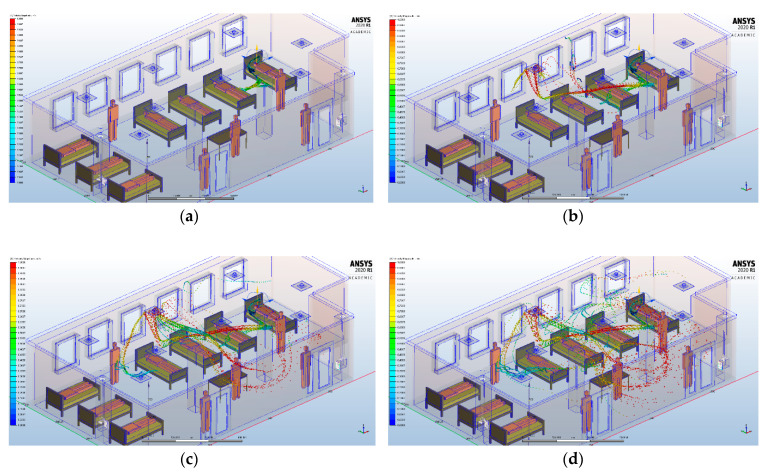
Transient analysis of the lighter particles dispersion carried into the air by buoyancy for HVAC systems, at different time intervals, if the infected patient is in the right part of the intensive care unit: (**a**) 2 s; (**b**) 3 s; (**c**) 4 s; (**d**) 5 s.

When the infected patient is on the right side of the intensive care unit and coughs, the droplets transmission is prompted by the HVAC system in the middle part of the room. We observe a fast transmission of the infecting particles, and after a period of only 5 s, they are spread almost in the entire intensive care unit (Figure 7). 

### 3.3. Scenario 2

**Figure 8:**
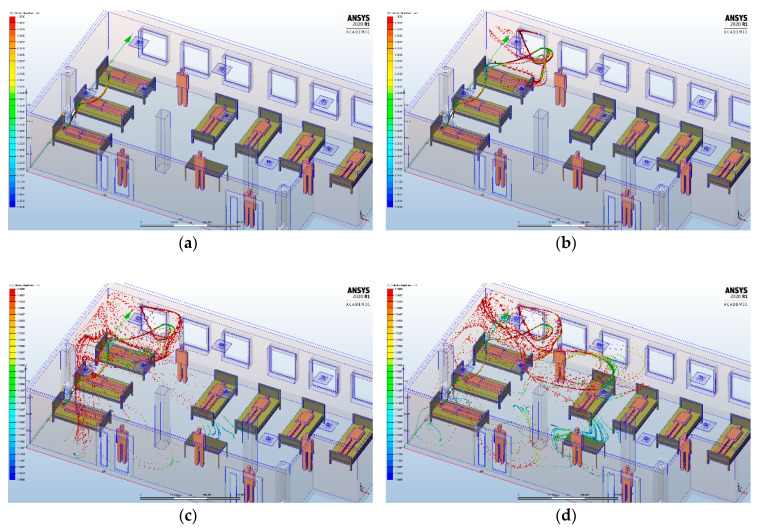
Transient analysis of the lighter particles dispersion carried into the air by buoyancy for HVAC systems, at different time intervals, if the infected patient is in the left part of the intensive care unit: (**a**) 2 s; (**b**) 3 s; (**c**) 4 s; (**d**) 5 s.

If the infected patient is on the left side of the intensive care unit and coughs, the droplets are also transmitted by the HVAC system in the central part of the room (Figure 8). Therefore, we observe that the HVAC system acts in the sense of a uniform distribution of the air currents, and consequently, of the infecting particles emitted by the patients’ cough. The fact that we did not notice a difference between the way the infecting particles are spread in these two scenarios shows that the HVAC system causes a rapid transmission, from the walls to the center, of the infecting particles. This is mainly influenced by the distribution of the outlets grids of the HVAC system, which in our case are located at the center of the intensive care unit.

### 3.4. Scenario 3

**Figure 9:**
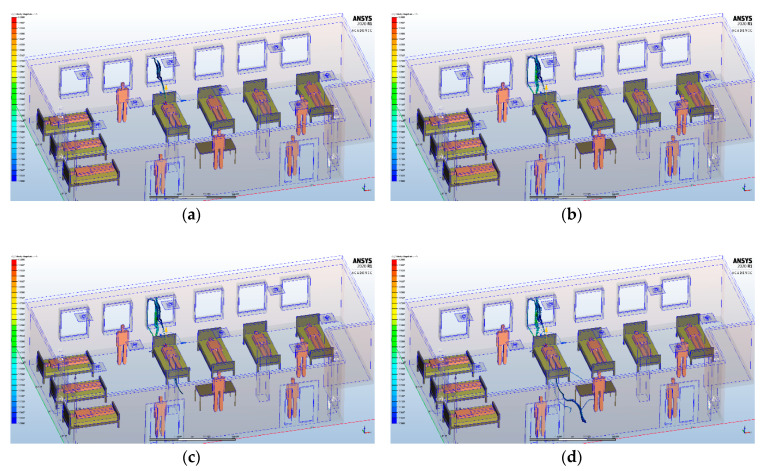
Transient analysis of the lighter particles dispersion carried into the air by buoyancy for HVAC systems, at different time intervals, if the infected patient is in the bed from the middle of the intensive care unit: (**a**) 2 s; (**b**) 3 s; (**c**) 4 s; (**d**) 5 s.

In the third scenario, we aimed to evaluate the infecting particle transmission if the patient is sitting in the bed from the middle of the intensive care unit (Figure 9). In case of this patient, considering that the inlet grid is located above the patient’s bed and also the fact that we simulated the patient’s cough almost in a vertical direction, determined a different transmission of the particles inside the intensive care unit. Thus, they were initially carried on the wall and the window behind the bed, then down under the bed, and after that to the office from the center of the intensive care unit. We can conclude that the presence of an HVAC system causes a distribution of infectious particles not only on directly exposed surfaces, but also on less exposed or hidden areas.

Comparing the results of the three proposed scenarios, it appears that, for our proposed HVAC system, the flow field and velocity distribution induced by the high turbulence of inlet grids, combined with the air outlet grids, determines wide recirculation zones, from the walls to the center of the room. These results could be due to the high rate level of ventilation. In the first two scenarios, we did not observe a difference between the way the infecting particles are spread, from the walls to the center of the intensive care unit. In the third scenario, it can be found that the location of the patient in the intensive care unit, the position of the inlet grid above the patient’s bed and also the fact that we simulated the patient’s cough almost in a vertical direction determined a different transmission of the infecting particles. However, the final path of the infecting particles was also to the center of the room. 

For the HVAC system used in our simulation, the variable directions of the airflow due to the high turbulence air inlet grids provide a widespread and a homogeneous distribution of the infecting particles (“coanda effect”) on the ceiling, followed by a progressive drop down. 

## 4. Discussion

In an intensive care unit, it is necessary to control the patient risk from airborne diseases, and the HVAC system can have a beneficial impact on patients’ healing processes and minimizing COVID-19 transmission. The present study highlights that CFD-FEM approach is useful for understanding the dispersion of infectious aerosols, and possibly of COVID-19-carrying droplets in a cardiac intensive care unit equipped with an HVAC system that has a variable air volume. The transient analysis of simulation results regarding particle tracing, paths and distance highlights the rapidity of the appearance of contaminated areas. Although it is known that a poor ventilation in confined indoor spaces increases the risk of respiratory infections transmission [48,49], we consider that the results from our CFD particle simulations have a great importance on the dispersion of infectious aerosols, and possibly of SARS-CoV-2 infection. The novelty of our study is that it provides useful indications for controlling dispersion and concentration zones for infectious aerosols and possibly of COVID-19-carrying droplets. Additionally, our results can provide important recommendations on the selection of the best position of the inlet and outlet grids. From our CFD simulations, we observed that it is very important to select a proper position for inlet and outlet grids, considering the fact that in scenario 2 the particles were dispersed more than those from the other two scenarios, which we did not expect. An explanation may be the onset of local turbulences that can cause an unpredictable particle dispersion. 

Only a few isolated cases of COVID-19 transmission events have been associated with closed spaces, also in asymptomatic patients [50,51], aided by air conditioning systems. A recent Chinese study pointed out the possibility of COVID-19 transmission, aided by air-conditioning systems in a restaurant. There were 10 cases from three families that have eaten lunch at the same restaurant. Those who were sitting along the line of airflow generated by the air-conditioning were infected, while those from other parts of the restaurant were not infected. They concluded that droplet transmission was facilitated by air-conditioned ventilation and the most important factor for infection was the direction of the airflow [52]. Additionally, two outbreaks in China have been described, where the authors suspected the air conditioning systems using a recirculating mode as a probable aid to COVID-19 transmission [53]. Thus, while the virus can be airborne, air-conditioning systems could aid virus transmission, under certain conditions such as airflow, improper filters or lack of ventilation. 

High Efficiency Particulate Air filters have demonstrated good performance with particles of the SARS Cov-2 virus size (approximately 70−120 nm) and are used in airplanes and in healthcare settings. Their important role in reducing the infection risk and in maintaining thermal comfort was demonstrated in a modeling study of the infection risk from SARS Cov-1 [54]. There are also some speculations regarding the use of air filtration technology (HEPA filters) in damping the rate of viral spread. Studies show that droplets typically expelled by infected patients, range from droplets that we are all able to see, to those too small for our eyes (0.5 to 15 microns). Additionally, studies of other viruses suggest that droplets of 1 micron are capable of carrying enough virus particles to cause infections [44]. HEPA filters capture particles of this size and also capture 99.97% of particles that are more than or equal to 0.3 microns in diameter. In theory, some experts say that considering the possibility of HEPA filters to capture particles of such small size, all SARS Cov-2 virions could be filtered and captured, in this way reducing the contamination of the space [31]. Thus, they propose installing HVAC systems with HEPA filters not only in the intensive care units, but also in high-traffic spaces, in order to decrease the number of viral particles present. 

On 3 April 2020, the Federation of European Heating, Ventilation and Air Conditioning Associations (REHVA), published the latest version of a guidance document where they summarize the advice on the operation and use of building services in order to prevent the spread of the COVID-19 [10].

1. Increase air supply in order to bring as much outside air as reasonably possible. Thus, their recommendation is to start ventilation at least two hours before the building usage time and switch to lower speed two hours after the building usage time, and keep the ventilation on 24/7, with lowered, but not switched off, ventilation rates when people are absent [10].
2. Humidification and air-conditioning have no effect in limiting the transmission of the virus, thus, they do not need to be adjusted. Coronaviruses are susceptible only to a humidity above 80% and a high temperature, above 30 ℃ [10]. Unfortunately, these parameters are not acceptable in buildings, because they will increase the microbial growth and will affect the indoor thermal comfort [10].
3. Another recommendation is to inspect the heat recovery devices considering the fact that maintenance personnel should follow the standard safety procedures of dusty work [10]. They also mention that when the HVAC system is equipped with a twin coil unit or another heat recovery device that completely separates the exhaust air side to the supply airside, virus particle transmission via heat recovery devices is no longer a problem. Regarding the use of recirculation, it is recommended to avoid central recirculation during SARS-CoV-2 episodes, in order to avoid resuspension of virus particles at room level [10].
4. Regarding the normal duct cleaning and maintenance procedures, no changes are needed and the most important two things are to increase fresh air supply and avoid recirculation of air [10].
5. Change of outdoor air filters or their replacement with other types of filters is not recommended sooner than normal [10].
6. Room air cleaners have a similar effect compared to ventilation and they remove particles from air. Devices that use electrostatic filtration principles for the supply or room air treatment may be useful [10].

Our study has some limitations and can be further improved. Firstly, this is only a numerical simulation study that evaluated the dispersion of infectious aerosols, and possibly of COVID-19 airborne transmission, based on the characteristics of SARS-CoV-2 and on the methods used in previous studies to evaluate airborne transmission for other infectious diseases. Further studies are needed in order to determine the impact of HVAC-systems on SARS-CoV-2 transmission in real cases. Secondly, the results of numerical simulations are only qualitative, by images, and we only considered exhaled droplets by coughing. Other investigations may consider quantitative results for other respiratory activities, such as breathing, sneezing or speaking. Thirdly, the composition of the infectious aerosols is of great significance in order to evaluate the infectious risk, and this deserves further study. Another limitation of our study is that we removed from our simulations the heavier particles, which usually fall to the ground, and we only focused on the lighter ones, which are carried into the air. This may be the reason why it seems that droplets air-dispersion is linear, rather than “clouds of droplets.” Additionally, the CFD characterization of the proposed HVAC system is more “case specific” for our cardiac intensive care unit. We are working now to find a more general CFD characterization that can be useful for the analysis of other targets, such as public transport, theaters, philharmonics and amphitheaters from educational units.

In an intensive care unit, it is necessary to have a negative pressure in order to reduce aerosol escape and also a high air-change rate to remove the infectious particles. Unfortunately, with the static heaters, these parameters cannot be achieved. By using an HVAC system, it is possible to create not only a negative pressure; in addition, we can prevent the transmission of the infectious particles outside the room when the medical staff use the door. Transmission of the infecting particles due to the air inlet grids is widespread and homogeneously distributed. This is counterbalanced by the possibility of using HEPA filters that are able to remove at least 90% of small particles, from inside and outside air. 

## 5. Conclusions

In conclusion, our study provides the mechanism for the dispersion of infectious aerosols, and the possibly of COVID-19 infection, by air conditioning systems. It also highlights important recommendations regarding the selection of the best position of the inlet and outlet grids in an HVAC system. Additionally, it can provide important recommendations for disease control and optimization of ventilation in intensive care units, by increasing the rate of air change, decreasing recirculation of air and increasing the use of outdoor air and HEPA filters. 

## Figures and Tables

**Table 1:** The boundary conditions used for computational fluid dynamics (CFD)-finite element method (FEM) simulations.

No.	Envelope Element	Heating System	
		Static Heaters	HVAC Heating System
CFD software—ANSYS FLUENT 2020 R1			
Turbulence model The RNG K-ε model			
Pressure (Pa)—Relative pressure			
Temperature (°C)	Exterior Walls	−16	−16
	Ceiling	+20	+20
	Input grids for hot air		+30
	Floor plate	+20	+20
	Windows and Doors	+18	+18
Heat transfer coefficient (film coefficient) U (W/m^2^ K)	Exterior walls	12	12
	Windows and doors	3	3
	Ceiling	3.5	3.5
	Floor plate	3	3
Air velocity(m/s)	Inlet grids for hot air	-	1.2
	Extraction grids	-	1
Heat flux (W/m^2^)	Static heater	200	-
Heat flow of mannequins	80 (W)		
Coughing velocity	12 m/s		
The total cough volume	1.1 dm³		
Mouth opening area	3.5 cm^2^		
Cough flow rate	5 dm³/s (L/s)		
Duration of one act of coughing	0.3 s		
The cough jet direction (supine) position	80°		
Temperature of the droplets	35 °C		
Max droplet diameter	200 µm		
Min droplet diameter	2.5 µm		
Particle cough density	2.5 µg/dm³		
Interaction between particles and walls-discrete phase conditions	Floor—trap; around—escape; inlet—mouth—escape; mannequin body—reflect; ceiling and walls—reflect.		
